# The outcomes of the modified Fulkerson osteotomy procedure to treat habitual patellar dislocation associated with high-grade trochlear dysplasia

**DOI:** 10.1186/s12891-017-1417-4

**Published:** 2017-02-08

**Authors:** Hong Chen, Daohong Zhao, Jingming Xie, Qihui Duan, Jun Zhang, Zhidan Wu, Jia Jiang

**Affiliations:** 1 Sports Medicine Department, the First People Hospital of Kun Ming City, Kunming, China; 2 Orthopaedic Department, the Second Affiliated Hospital of Kun Ming Medical University, Dian Mian Road 374, Kunming, China; 3 Sports MedicineDepartment, Huashan Hospital, Fudan University, Shanghai, China

## Abstract

**Background:**

Habitual patellar dislocation is not common in clinical practice, but it has a deep impact on the patient's lifestyle and movement. There has been no large case-control study on habitual patellar dislocation, and the management of it is still controversial.

The aim of this study was to observe the efficacy of the modified Fulkerson procedure on patients with habitual patellar dislocation with high-grade trochlear dysplasia without trochleoplasty and to evaluate the results of this procedure.

**Methods:**

A total of 25 patients who were admitted to our hospital from April 2007 to October 2013 were included: 7 males and 18 females, aged 17–28 years old, with an average age of 21.5 years old, including 21 cases of unilateral dislocation and 4 cases of bilateral dislocation. The tibial tuberosity transfer procedure (internal rotation, medial transfer and elevation osteotomy) and medial patellofemoral ligament (MPFL) reconstruction were performed in all cases of habitual patellar dislocation that were accompanied by trochlea dysplasia.

**Results:**

The mean follow-up duration was 36.8 months (range, 25–68 months). A CT scan was performed to compare the tibial tuberosity–trochlear groove distance (TT-TG), the patellar tilt angle (PTA), and the mean Kujala and Lysholm scores before surgery and at follow-up and to measure the angle of internal rotation of the tibial tubercle after surgery. The mean Kujala and Lysholm scores improved significantly (*P* < 0.05) from 55.65 ± 6.10 and 50.34 ± 6.54 preoperatively to89.24 ± 4.66 and 88.53 ± 4.75, respectively, at follow-up. The tibial tuberosity–trochlear groove distance (TT-TG) decreased significantly (*P* < 0.05) from 20.24 ± 2.80 mm to 10.50 ± 4.50 mm, and the patellar tilt angle (PTA) decreased significantly (*P* < 0.05) from28.58 ± 3.28to7.54 ± 5.56. No recurrence was observed, and only one patient had a mild skin infection after surgery. The mean angle of internal rotation of the tibial tubercle was 10 ± 4° after surgery. There were no cases of stiffness.

**Conclusions:**

The modified procedure of tibial tubercle transfer, especially the internal rotation, which can improve the patella stability and knee function, is an effective surgical procedure for the treatment of habitual patellar dislocation associated with high-grade trochlear dysplasia without trochleoplasty.

**Level of evidence:**

III

## Background

Habitual patellar dislocation is rare in the clinic and often occurs during early childhood. There is still much controversy associated with aetiological factors, pathological changes and treatment, especially in the choice of surgical method [1]. There are many traditional treatments, including proximal and distal realignment and soft tissue procedures, which lead to a high rate of recurrence and an increased incidence of osteoarthritis in the patellofemoral joint postoperatively at long-term follow-up [2]. Trochlear dysplasia is one of the most consistent anatomic factors that can lead to habitual patellar dislocations. Various trochleoplasty procedures have recently been described to treat patellar dislocation, but none of them can be popularized because they involve a complex operation and complications, and long-term follow-up is lacking [3]. There has been no standard procedure until now.

In this study, we hypothesized that the modified Fulkerson osteotomy procedure would be efficient to treat patients with habitual patellar dislocation associated with high-grade trochlear dysplasia without trochleoplasty, and our purpose was to evaluate the results of this procedure and to observe the efficacy of the procedure.

## Methods

This study was performed according to a protocol that was approved by the Institutional Review Board of Kun Ming Medical University. All subjects provided their informed consent to participate in this study. A total of 25 patients (29knees) were included from April 2007 to October 2013, with a mean patient age of 21.5 years (range, 17–28 years), including 21 cases of unilateral dislocation and 4 cases of bilateral dislocation; the dislocation reappeared during flexion in each patient. Inclusion criteria for the study: patients with a habitual patellar dislocation (dislocation can occur during each flexion) with high-grade trochlear dysplasia type B,C and D according to the Dejour classification [4] and no previous patellofemoral surgery. Patients with open growth plates, patellofemoral arthritis, or patellofemoral pain syndrome with no true dislocation were excluded from the study.

The clinical examination included the reappearance of the dislocation and confirmed trochlear dysplasia as detected via preoperative CT scans. According to the Dejour classification, there were 11knees of type B, 14 knees of type C, and 4 knees of type D. The tibial tuberosity–trochlear groove distance (TT-TG) was greater than 20 mm in 17 knees and was between 13 mm and 20 mm in 12 knees. The patellar position (Caton-Deschamps index), as measured via CT scan, was normal.

### Surgical technique

All of the patients in the present study were treated by the same senior doctor. Epidural anaesthesia or general anaesthesia and standard arthroscopic portals were established and a thorough arthroscopic evaluation of the joint was performed, which was used to determine whether the cartilage, meniscus or ligament was injured. The patellofemoral joint congruence and the development of the trochlear sulcus were evaluated under arthroscopy through the superior lateral patellar approach. An incision of approximately 10 cm was made in the midline of the knee from the above patella to the anterior tibial tubercle. The lateral retinacula was evaluated and released, the lateral head of the quadriceps was cut off, and the shrinkage of the iliotibial band was cut off at the same time. An attempt was made to keep the integrity of the capsule. According to the patellar tilt angle, we determined whether to perform a medial patellofemoral ligament (MPFL) reconstruction by using the autologous semitendinosus anatomic double-bundle method. Tibial tubercle osteotomy: the modified Fulkerson procedure was performed. The patellar tendon was exposed, then a K-wire was used to drill intermittently on the preplanned osteotomy line around the tibial tubercle, and an osteotomy was used to dissociate the tibial tubercle from the inside to the outside at 45° like an inverted triangle. The length of the graft was approximately 5 cm. The medial part of the bone, which was inside the cutting bed, was also removed. According to the TT-TG values that were measured prior to the operation, a dissociated-tibial tubercle medial transfer was performed to 10-15 mm as normal, and simultaneously, the tibial tubercle was internally rotated. The extent of internal rotation was adjusted intraoperatively according to the developmental condition of the femoral condyle so that the patella could bypass the lateral eminence of the femoral condyle during the knee flexion process. Without causing a high patella, the tibial tubercle was elevated appropriately during surgery. The tibial tubercle was temporarily fixed with K-wire. After checking the stability of the patellofemoral joint and the motion trail of the patella, we finally fixed the tibial tubercle with 2–3 cortical bone screws in different directions. We observedthe involution of the patellofemoral joint and the patellar tilt angle (PTA) again to ensure a good involution for the patellofemoral joint, a significantly decreased angle of patellar tilt and a reduced pressure of the medial patellofemoral joint. We examined the knee again to ensure that it was normal, without snapping or dislocation, and to verify that the motion trail of the patella was normal.

### Postoperative process

After surgery, the knee was placed in a hinged knee brace. Exercises such as ankle pumping, quadriceps strengthening, straight leg raising, and patella pushing were performed as tolerated. Gradual passive and active ranges of motion were achieved on the 7th day after surgery. Patients were allowed to flex at 90° on the 3rd to the 4th week after surgery, and weight-bearing as tolerated with a hinged brace was achieved on the 6th to the 8th week after surgery. On the 3rd month after surgery, mild jogging and partial sports activities were recommended.

Follow-up Indicators: The mean follow-up duration was 36.8 months (range, 25–68 months). Complications and outcomes were recorded at the time of follow-up. After checking the stability of the patella, the author recorded the number of cases of dislocation that occurred. The patellar tilt angle (PTA) was measured postoperatively via CT scanning at 20°of knee flexion. The Lysholm and Kujala scoring systems were used for the clinical assessments of the results. The tibial tuberosity–trochlear groove distance (TT-TG), the angle of internal rotation of the tibial tubercle and the patellar tilt angle (PTA) were measured by CT scan at 20° of knee flexion.

Data were processed with SPSS 19.0 statistical software. The values of the preoperative and postoperative data were compared using the paired *t*-test. Statistical significance was set at *P* < 0.01.

## Results

**Fig. 1:**
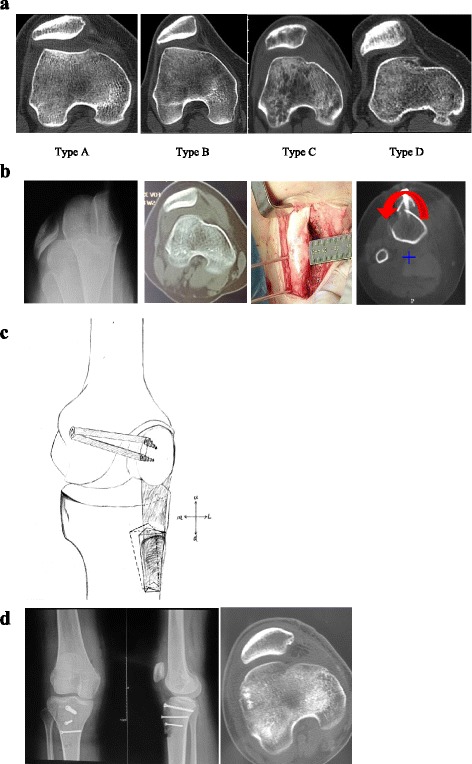
Female, 17 years old, habitual patellar dislocation of the left knee. **a** Dejour type oftrochlear dysplasia. Type A dysplasia: trochlear morphologic structures are preserved, but the sulcus is shallow. Type B dysplasia: flat, horizontally oriented trochlear joint surface. Type C dysplasia: flat, obliquely oriented trochlear joint surface with facet asymmetry. Type D dysplasia: same as type C but with a prominent bone protrusion. **b** CT scan image showingtrochlear dysplasia type C; tunnel view showing thepatellar dislocation; osteotomy of tibial tuberosity (+: tibial tubercle;  : internal rotation and medial transfer). **c** Schematic diagram of the elevation of the tibial tubercle and MPFL reconstruction. **d** Two years after the operation, the X-ray and CT images show a normal patellar position

The mean Kujala and Lysholm scores improved significantly from 55. 65 ± 6.10 and 50.34 ± 6.54 preoperatively to 89.24 ± 4.66 and 88.53 ± 4.75, respectively,at the time of follow-up. The tibial tuberosity–trochlear groove distance (TT-TG) decreased significantly from 20.24 ± 2.80 mm to 10.50 ± 4.50 mm, and the patellar tilt angle (PTA) decreased significantly (*P* < 0.05) from 28.58 ± 3.28to7.54 ± 5.56 (Table 1). No recurrence was observed, and only one patient had a mild skin infection after surgery, which healed after being positively handled. The mean angle of pronation of the tibial tubercle was 10 ± 4° after surgery (Fig. 1). At the last follow-up, there were no recurrences of patellar dislocation and no cases of stiffness.

**Table 1:** Comparison of the results of clinical follow-up after surgery (x ± s)

Time	Kujala score	Lysholm score	TT-TG (mm)	PTA
Pre-op	55. 65 ± 6. 10	50.34 ± 6.54	20.24 ± 2.80	28.58 ± 3.28
Post-op	89.24 ± 4.66	88.53 ± 4.75	10.50 ± 4.50	7.54 ± 5.56
T value	−41.235	−43.155	13.432	28.85
P value	<0.01	<0.01	<0.01	<0.01

## Discussion

Patellar dislocation may be classified into congenital, recurrent and habitual. Habitual patellar dislocationis rare and often occurs during early childhood. Part of a recurrent patellar dislocation could develop into a habitual dislocation when the patellar dislocation occurs due to an external injury of the knee joint [5]. In agreement with most of the previous studies [5, 6], the cases in this group consisted of patients whose knee patella was in the normal position when the knees were unbent and dislocated laterally when the knees were bent past 30° [6]. Nevertheless, there were some opposing reports that the knee patella was located in the normal position when the knees were bent and were laterally dislocated when the knees were unbent. Some patients may suffer from bilateral dislocation, and there were 4 patients with bilateral dislocation in our study. Most of the habitual patellar dislocation patients suffer with knee joint dysplasia, but its pathogenesis factor is not exactly clear. The pathological changes that were observed in this paper mainly included: (1) weak medial structures; (2) lateral structure contracture;(3) femoral trochlear dysplasia;(4) valgus deformity, tibia extortion. At present, most scholars advocate for surgery at the early stage, which could normalize the development of the trochlear, and avoid the occurrence of advanced osteoarthritis at the same time. However, the treatments are numerous, andsurgery remains in dispute. We think that the pathological changes should be the basis of a surgical choice.

Recently, in the studies of the medial patellofemoral complex, most authors agree that the MPFL plays a major role in limiting the process to prevent the lateral dislocation of the patellar [7, 8]. However, most patients exhibited habitual patellar dislocation with high-grade trochlear dysplasia, which could not correct the abnormal bone structure [9–11]. Patellar dislocation is often caused by a variety of abnormal anatomic factors. When the Q angle or thetibial tuberosity–trochlear groove distance (TT-TG) increases, the ligament would be under too much tension, which would greatly increase the risk of failure long-term if only a medial patella-femoral ligament reconstruction was performed [12]. By shifting the tibial tubercle and effectively adjusting the motion trail of the patella, lowering the tibial tuberosity–trochlear groove distance (TT-TG) and reducing the tension of medial patellofemoral ligament, there is also a corresponding stress reduction on the patellofemoral joint [13].

Traditional tibial tubercle osteotomy was mainly used to adjust the distance of the tibial tuberosity–trochlear groove and the location of the patella. The use of a modified Fulkerson osteotomy can meet the demand of medial transfer, internal rotation and elevation osteotomy. For this group of patients, a femoral condyle “bulge” and medial condyle dysplasia existed. During the flexion of the knee, the patella femoral condyle often leads to dislocation because the lateral femoral condyle eminence cannot be bypassed. Therefore, in theory, a trochleoplasty should be performed. Recently, many studies have reported that trochleoplasty was only applied topatients with trochlear dysplasia typesB and D [14, 15], but the majority of the patients in this study were type C (14 knees) in this study. Additionally, in a long-term follow-up of patients who had undergone trochleoplasty, patients faced cartilage necrosis, severe patellofemoral arthritis, joint adhesions and other complications [16]. On the other hand, most cases of trochlear dysplasia are often combined with patella dysplasia. Therefore, the exclusive use of trochleoplasty was inadequate.

A studyby Von Knoch [17] et al. that included 45 patients who had been treated with trochleoplasty for recurrent patellar dislocation demonstrated that 30% of patients reported increased patella-femoral pain that was associated with the presence of a degenerative knee at long-term follow-up. Verdonk [18] et al. reported that 13 patients had undergone trochleoplasty for patellar instability or patellofemoral pain. Good results were reported for 46% of patients in subjective postoperative evaluations, but the results of other patients were moderateor poor according to the objective scoring. Through the internal rotation of the tibial tuberosity, the patella could directly bypass the lateral femoral condyle, which had beneficial effects on patellar dislocation during the inflexion motion of the knee. Meanwhile, the tilt angle of the patella was adjusted, which increased matching of the patellofemoral joint, especially for femoral condyle dysplasia patients. A total of 25 patients in this group were determined to exhibit a good effect, which ensured a good motion trail of the patellofemoral joint, and reduced the stress of the MPFL.

Patellofemoral arthritis has been a common problem that affects long-term postoperative outcomes [19]. All of the patients in our study exhibited recurrent patellar dislocation, and there was no case with a high or low position of the patella according to the Caton-Deschamps index and the preoperative CT images. During the operation, we simultaneously preformed tibial tubercle elevation to appropriately reduce the patellofemoral joint stress and to avoid serious complications, such as patellofemoral arthritis, so all patients were painless and had no radiographic signs related to patellofemoral arthritis at follow-up.

Admittedly, there are many shortcomings in this study: an insufficient sample size and a short follow-up time; no case-control study on different surgical methods; the tibial tubercle osteotomy only applies to patients with complete skeletal development; radiographic evaluation was exclusively used for the postoperative patellofemoral joint; no second microscopy was performed to further evaluate the cartilage. In conclusion, in theory, trochleoplasty should be applied to patients with trochlear dysplasia; however, it is not extensively used because the surgical technique is demanding and long-term clinical follow-up is lacking. We adjusted the patellofemoral congruence with osteotomy on the tibial tubercle, especially on the internal rotated tibial tubercle, to overcome the impact of trochlear dysplasia on dislocation. In our view, the procedure of tibial tubercle transfer, especially the internal rotation procedure, is a useful surgical modality for the treatment of habitual patellar dislocation associated with trochlear dysplasia without trochleoplasty.

## Conclusions

The modified procedure of tibial tubercle transfer, especially internal rotation, is an effective surgical procedure for the treatment of patients with habitual patellar dislocation associated with high-grade trochlear dysplasia without trochleoplasty, and can improve patella stability and knee function.

## Abbreviations

MPFL: Medial patellafemoral ligament
TT-TG: Tuberosity–trochlear groove distance
PTA: Patellar tilt angle
